# Functionalization-Driven Formation of TiO_2_/Ti_2_CT_*x*_ Interfaces

**DOI:** 10.1021/acs.jpcc.5c04505

**Published:** 2025-10-17

**Authors:** Néstor García-Romeral, Giovanni Di Liberto, Ángel Morales-García, Francesc Viñes, Francesc Illas, Gianfranco Pacchioni

**Affiliations:** † Departament de Ciència de Materials i Química Física & Institut de Química Teòrica i Computacional (IQTCUB), Universitat de Barcelona, c/Martí i Franquès 1-11, 08028 Barcelona, Spain; ‡ Dipartimento di Scienza dei Materiali, Università di Milano−Bicocca, via R. Cozzi 55, 20125 Milano, Italy

## Abstract

In this study, the nature of the interface between TiO_2_ and MXenes was systematically explored by using density functional theory and taking Ti_2_C as a case study. TiO_2_/MXene interfaces are emerging as potential photoactive systems, but a deep understanding of their nature is often elusive. Our findings reveal that MXene surface functionalization predominantly governs the interaction between TiO_2_ and MXenes. Specifically, when the Ti_2_C MXene is terminated with −H or −OH or when it is not functionalized, the formation of the interface leads to a strong interaction due to the formation of new chemical bonds. Weak van der Waals interactions are present when the Ti_2_C MXene surface termination involves −F, −Cl, or −O groups. The analysis of the interface polarization shows a systematic charge transfer from MXene surfaces toward TiO_2_, which is localized at the interface and influenced by MXene functionalization. The results of this study provide new atomistic insights on the interaction between these two materials, helping the understanding of the nature of this emerging class of photoactive materials.

## Graphical abstract

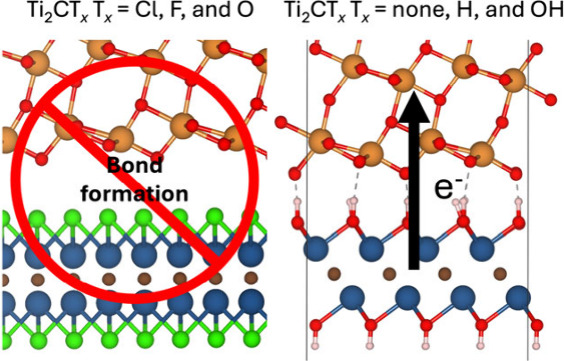

## 1 Introduction

Titanium oxide (TiO_2_) is a widespread semiconductor with different potential applications that involve the use of light in chemical processes, such as photocatalysis. This puts TiO_2_ in a privileged position for energy and environmental remediation.^1^ However, nanostructured TiO_2_ materials made by either anatase (A-TiO_2_) or rutile TiO_2_ (R-TiO_2_), the two main polymorphs, face challenges that hinder their potential in photocatalysis. The main issue is its wide band gap of 3.0–3.2 eV (depending on the specific polymorph), which restricts the ability to capture sunlight photons with energy in the visible range.^2^ A second equally important problem is related to the rapid recombination of photogenerated electron–hole pairs. The short lifetime of these charge carriers and their high recombination rate significantly reduce the efficiency of photocatalytic reactions.^3,4^

To address these limitations, a series of strategies have been extensively developed to improve the photocatalytic performance of TiO_2_. These include doping TiO_2_ with metal atoms (Ag, Au, Pd, Pt, and Ru) or nonmetal atoms (C and N),^5–10^ modifying the morphology and size of TiO_2_ nanoparticles,^2,11–13^ or interfacing TiO_2_ with other materials to create heterostructures.^14–21^ These strategies allow promoting the excitations of hole and electrons in the visible range and/or long-lived photogenerated carriers due to spatial electron–hole separation.^22^

Applying the strategy of creating heterostructures, the combination of TiO_2_ with MXenes is quickly emerging. MXenes are a relatively new class of few-layered two-dimensional (2D) materials consisting of metal carbides, nitrides, or carbonitrides,^23–25^ typically featuring surface termination groups. MXenes can be synthesized by means of a selective chemical etching procedure of MAX phases, which have M_*n*+1_AX_*n*_ chemical formula, where M represents an early transition metal, A denotes elements from groups XIII or XIV, X is C and/or N, and *n* ranges from 1 to 4, determining the MXene thickness.^26–28^ The resulting MXene has M_*n*+1_X_*n*_T_*x*_ as a formula, where T_*x*_ denotes chemical groups (or functionalization groups) attached to the MXene (0001) surfaces due to synthetic conditions, usually −Cl, −F, −H, −O, and −OH.^24,25^ The variety of the chemical agents employed in the etching procedure and the experimental conditions affects the MXene termination. For instance, the −F termination can be found when HF or *in situ* acid is used as etchant.^29^ Other synthetic fluorine-free routes are possible. For instance, the use of HCl acid can lead to −Cl terminations.^30^ In addition, a postsynthesis hydrothermal process can be performed, leading to −O, −H, and −OH terminations.^31^ Additional steps can promote efficient removal of these T_*x*_ groups,^32,33^ resulting in bare MXene (0001) surfaces, M_*n*+1_X_*n*_.^24,32,34^

TiO_2_/MXene heterostructures are normally obtained by oxidation of corresponding Ti-based MXene surfaces in aqueous environments, which leads to the formation of the TiO_2_/Ti_*n*+1_X_*n*_T_*x*_ heterostructures.^35,36^ It is worth noting that this oxidation process can be experimentally controlled to obtain TiO_2_/Ti_*n*+1_X_*n*_T_*x*_ interfaces with different TiO_2_ polymorphs and surfaces.^37^ These partially oxidized TiO_2_/Ti_*n*+1_X_*n*_T_*x*_ heterostructures have proven their potential as photocatalysts for several key chemical processes such as CO_2_ photoreduction,^38–40^ H_2_ generation,^41–44^ and N_2_ fixation.^45,46^ These studies have revealed that TiO_2_/Ti_*n*+1_X_*n*_T_*x*_ heterostructures promote efficient electron–hole separation while maintaining the redox capabilities of charge carriers.^37,47^ The separation is driven by the high electrical conductivity of MXene materials.^48^ The presence of a Schottky barrier at the metal–semiconductor interface prevents the recombination of the photoinduced charge carriers, blocking the reverse flow of electrons from the MXene toward the TiO_2_.^37,47,49,50^ From the theoretical side, Xu *et al*.^51^ used the Perdew–Burke–Ernzerhof (PBE)^52^ functional to investigate the interfacial properties of TiO_2_/Ti_*n*+1_C_*n*_T_*x*_ (*n* = 1 and 2) heterostructures including a few −F, −O, and −OH terminations and focused on the (101) surface to represent the slab model of A-TiO_2_. In addition, nanostructured (TiO_2_)_5_ and (TiO_2_)_10_ clusters have been supported on the bare Ti_2_C, and (TiO_2_)_5_, on various M_2_C­(0001) surfaces, with M = Ti, Zr, Hf, V, Nb, Ta, Cr, Mo, and W, showing a remarkable exothermic interaction of MXenes coupled to a considerable charge transfer from the bare MXene surface toward the TiO_2_ clusters.^53,54^ In addition, the effect of the Ti_2_C MXene functionalization on the formation of these composites has also been studied.^55^

Despite the vast attention to these heterostructured systems, atomistic understanding by means of quantum chemical approaches still must address a series of key points. First, MXene surfaces are usually functionalized (T_*x*_) with −F, −Cl, −O, −H, and −OH groups,^24,25^ and their role in the TiO_2_/Ti_*n*+1_X_*n*_T_*x*_ interface may have a critical influence. Second, the nature of the TiO_2_ face is equally important in principle.^56,57^ Thus, the aim of this work is to systematically investigate, by means of first-principles calculations, the effect of different polymorphs, the surface exposure of TiO_2_, and the effect of the T_*x*_ termination in the interaction between TiO_2_ and Ti-based MXenes. We focus on Ti_2_CT_*x*_ following previous results by some of us.^58^ Our results will provide atomistic insights into the nature of these interfaces, revealing that the interaction is mainly governed by MXene surface functionalization. In just a few cases, it is possible to have interfaces where new chemical bonds are formed. The results of this study are expected to contribute to the fundamental understanding of these objects and help in their design and optimization.

## 2 Models and Computational Details

### 2.1 Models

To build TiO_2_/Ti_2_CT_*x*_ interfaces, we considered the most common A-TiO_2_(101) and (101) and R-TiO_2_(110) surfaces along with Ti_2_CT_*x*_(0001) where T_*x*_ = Cl, F, H, O, OH, and an unfunctionalized surface, *aka* “none”. In the case of A-TiO_2_, we optimized the bulk crystal structure and then created slabs along the (001) and (101) directions. Next, the atomic coordinates were fully optimized. The same procedure was applied for R-TiO_2_ preparation of the (110) slab. Figure 1a–c shows the optimized model of each TiO_2_ phase. Table S1 in the Supporting Information reports the relevant quantities, such as the *a* and *b* lattice parameters, the surface energy (γ), the slab thickness (*d*), and the total number of atoms of each slab model. Note that given the employment of *p*(1 × 1) unit cells for A-TiO_2_ and R-TiO_2_ surfaces, and conventional *c*(1 × 1) unit cells in Ti_2_CT_*x*_ MXene models, the angle between *a* and *b* is always 90°. Previous studies showed that the thickness of the present TiO_2_ surface models is sufficient to reasonably represent surface phenomena.^59,60^

**Figure 1:**
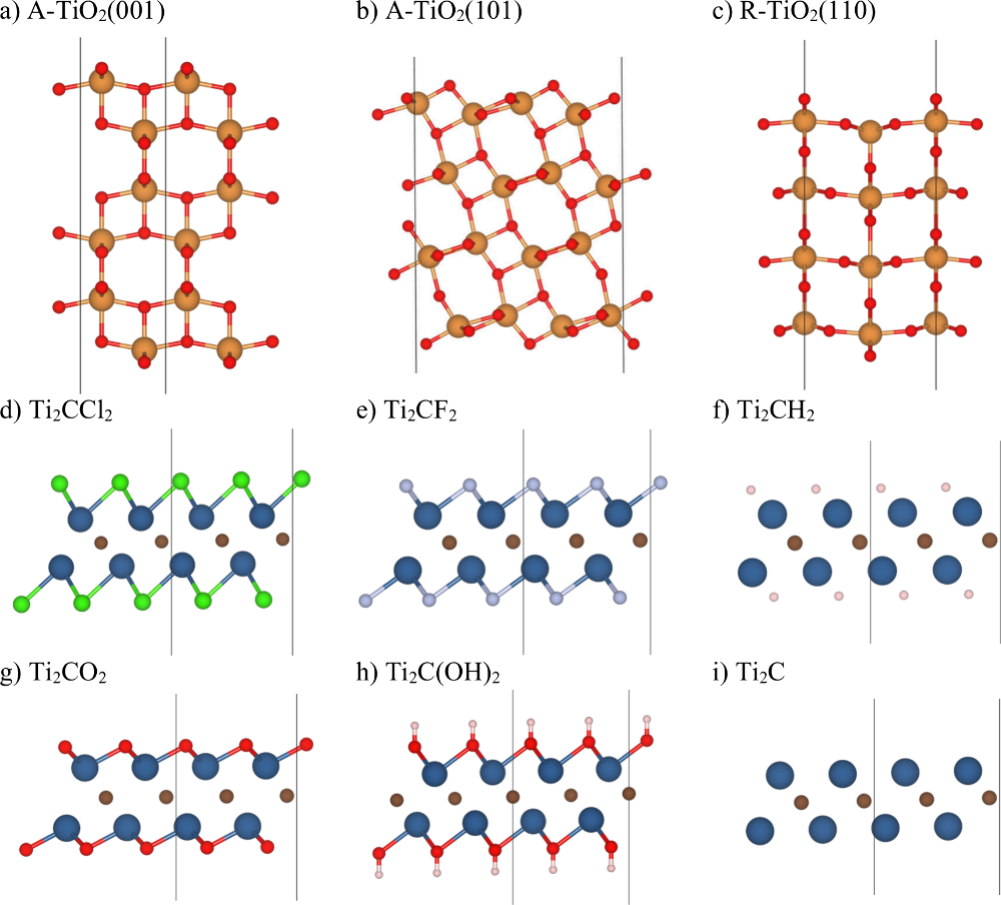
Side views of optimized geometries of *p*(1 × 1) for (a) A-TiO_2_(001), (b) A-TiO_2_(101), and (c) R-TiO_2_(110) and conventional *c*(1 × 1) for (d) Ti_2_CCl_2_, (e) Ti_2_CF_2_, (f) Ti_2_CH_2_, (g) Ti_2_CO_2_, (h) Ti_2_C­(OH)_2_, and (i) Ti_2_C. Dark lines represent the unit cell borders. Orange and red spheres represent the Ti and O atoms from TiO_2_, respectively, while blue, brown, red, and pale pink spheres represent the Ti, C, O, and H atoms from Ti_2_CT_*x*_, respectively.

Next, in order to model all functionalized Ti_2_CT_*x*_ MXenes, the conventional *c*(1 × 1) cell of pristine ABC-stacked Ti_2_C was employed. This MXene was functionalized with Cl, F, H, O, and OH chemical groups on each hollow metal site, the most favorable adsorption site according to previous studies, of the bare Ti_2_C on both (0001) surfaces, leading to a CABCA stacking.^61,62^ Figure 1d–i presents the optimized model of each MXene termination. Table S1 reports structural information for all studied MXenes. The main purpose of this study is to examine how the functional groups present on the MXene surface influence their interaction with TiO_2_. To address this problem, we adopted model systems where the MXenes are defect free and are fully covered by their respective functional groups. It must be mentioned that coverage could have an effect that goes beyond the purpose of this study. This will be considered in a future work. We considered defect free models to focus on the interface properties depending on the MXene termination. Of course, defects can be present in real samples as is well documented for TiO_2_.^63–65^ The effect of defects on the interface properties is a challenging aspect and is generally system dependent. Indeed, it could have important implications or be negligible depending on the specific system. For instance, some of us investigated the nature of SrTiO_3_/TiO_2_ finding a secondary effect induced by oxygen vacancies located either in the interface region or in the bulk of the materials.^66^

Once the TiO_2_ slabs and Ti_2_CT_*x*_ surfaces models were obtained, a series of heterostructures with the help of the VASPKIT program were generated.^67^ The interfaces were prepared to have the smallest possible mismatch of lattice parameters to avoid spurious effects. Typically, an acceptable trade-off between mismatch of lattice parameters and number of atoms in the cell content is at most 3%.^68^ However, the effect of mismatch always needs to be checked case by case. We tested this effect on the electronic properties of each building block, results of which are reported in Tables S2 and S3. The results show a negligible effect on the electronic properties. Therefore, the choice of the working cells can be considered to be acceptable for the purpose of the study. Indeed, we checked for the presence of spurious effects to the band gap or Fermi energy of semiconductor and conductive objects, respectively (see Tables S2 and S3) with the PBE functional. Changes within 0.1 eV are found, which can be considered an acceptable result as it is within the accuracy of the approach.^69,70^ The total number of atoms for each supercell is reported in Table S2, where the number of atoms in the simulation box is in the range 200–400 atoms.

### 2.2 Computational Details

We performed all periodic calculations using Density Functional Theory (DFT) as implemented in the Vienna *Ab Initio* Simulation Package (VASP).^71,72^ The Kohn–Sham equations were numerically solved employing a plane wave basis set to depict the valence electron density with a working kinetic energy cutoff of 415 eV. The interaction between the core and valence electron densities was accounted for by the Projector Augmented Wave (PAW) method.^73^ To describe the exchange correlation term of the density functional for the optimization of the models, the Generalized Gradient Approximation (GGA) was used within the Perdew–Burke–Ernzerhof (PBE) parametrization.^52^ This functional is commonly used to study materials, due to its robustness, low computational cost, and overall good performance with acceptable accuracy in the description of metallic systems,^74–78^ such as bare MXenes. In addition, we performed test calculations and optimized the crystal structure of *c*(1 × 1) Ti_2_C with the Heyd–Scuseria–Ernzerhof (HSE06) functional to check for the effect of relaxation with hybrids. A maximum change of 0.06 Å of the lattice parameters was found. For this reason, we decided to work with single-point HSE06 calculations to refine the electronic structure, avoiding energy intensive geometry optimizations. On the other hand, it faces some drawbacks in reproducing the semiconducting character of materials, with an underestimation of the band gap. Nevertheless, it often provides acceptable results for other properties, such as the structure and role of quantum confinement.^79^

A possible solution to overcome the limitation of the PBE functional is to invoke hybrid functionals, which are more computationally expensive, especially when using a plane wave basis set. A way to attenuate the drawback with acceptable accuracy to carry out energy and electronic structure analyses is to perform single-point calculations with a hybrid functional on top of the PBE relaxed structures. We selected the hybrid HSE06 functional.^80^ This hybrid functional is efficient for closed-shell, metallic, and magnetic systems. Therefore, the geometry and structural analysis relies on the PBE functional, and results of the electronic structure and energetics are obtained with the HSE06 functional. The total DOS of each isolated building block and the projected DOS on each building block for each heterostructure are reported in Figure S1. First, focusing on isolated TiO_2_ phases, all show semiconducting behavior, with energy band gaps of 3.4, 3.9, and 3.6 eV for A-TiO_2_(001), A-TiO_2_(101), and R-TiO_2_(110) surfaces, respectively (see Figure S1a–c).^56^ As is well-known, we find that HSE06 tends to slightly overestimate the gap of TiO_2_ compared to the experimental values, *i.e.*, 3.2–3.4 eV for A-TiO_2_ and 3.0 eV for R-TiO_2_.^2^ On the other hand, the Ti_2_C, Ti_2_CH_2_, and Ti_2_C­(OH)_2_ MXenes show gapless electronic structures, denoting a general metallic character, in agreement with previous studies.^81,82^ The different MXene functionalization has an impact on the work function of the MXene (0001) surface, leading to different Fermi energy levels as shown in Figure S1d–f.

These differences between the work functions of TiO_2_ phases and MXene surfaces may lead to a charge transfer at the interface. Since all MXene work functions are lower than the ones of all TiO_2_ phases, it is expected that the charge transfer takes place from Ti_2_CT_*x*_ (T_*x*_ = none, H, and OH) toward TiO_2_ phases regardless of the MXene functionalization and TiO_2_ phase, in agreement with the below discussed results of interface polarization.

Since dispersion interactions between the TiO_2_ and Ti_2_CT_*x*_ surfaces may play a role, these are accounted for in all calculations by means of the Grimme’s D3 method with the Becke–Johnson damping function approach.^83^ In order to avoid artificial interactions between replicas, a vacuum space of 18 Å along the direction normal to the MXene (0001) surface (*z* direction) was included. Given the large size of the heterostructure supercells, all calculations were carried out at the **Γ** **k**-point only. On the other hand, for bulk and surface systems, we performed test calculations at the *c*(1 × 1) Ti_2_C cell to check for the reliability of the working sampling of the reciprocal space (see Table S4). We decided to work employing 3 × 3 × 1 **k**-points grid.^84^ The convergence threshold for the electronic Self-Consistent Field (SCF) calculations was set at 10^–5^ eV, and the structural optimizations, including lattice parameters, were reached when atomic forces were below 10^–2^ eV/Å.

All calculations were performed without considering spin-polarization of the electronic density to save computational time. However, postprocess, possible errors were checked on a few cases, by comparing with spin-polarized calculations. Table S5 compares the results for the A-TiO_2_(101)/Ti_2_C system, where the MXene is not functionalized. This is probably the most delicate case to test as when the Ti_2_C­(0001) surface is functionalized, the inherent magnetism of Ti_2_C is removed.^82,85^ These results show that when the electronic shell is open, there is no significant change in the interaction strength values and properties of the heterostructure.

The electronic interaction strength of TiO_2_/Ti_2_CT_*x*_ heterostructures was measured by using as a proxy the interface energy defined as

1 $$E_{\mathrm{int}}=\left(E_{\mathrm{Ti}O_{2}/{\mathrm{Ti}}_{2}CT_{x}}-E_{\mathrm{Ti}O_{2}}^{\mathrm{sp}}-E_{{\mathrm{Ti}}_{2}CT_{x}}^{\mathrm{sp}}\right)/A$$

where *E*_TiO_2_/Ti_2_CT_*x*__ is the total energy of the optimized heterostructure and *E*_TiO_2__^sp^ and *E*_Ti_2_CT_*x*__^sp^ are the single-point (sp) total energies of each isolated building block at the interface geometry. *A* is the contact area in the optimized heterostructure. Next, the interface polarization was determined by means of the Charge Density Difference (CDD), Δρ, and the Plane-Averaged CCD (PA-CCD), along the normal direction of the Ti_2_CT_*x*_ (0001) surface (the *z* direction), eq 2.

2 $$\Delta \rho =\rho_{\mathrm{Ti}O_{2}/{\mathrm{Ti}}_{2}CT_{x}}-\rho_{\mathrm{Ti}O_{2}}^{\mathrm{sp}}-\rho_{{\mathrm{Ti}}_{2}CT_{x}}^{\mathrm{sp}}$$

where ρ_TiO_2_/Ti_2_CT_*x*__, ρ_TiO_2__^sp^, and ρ_Ti_2_CT_*x*__^sp^ are the charge densities of TiO_2_/Ti_2_CT_*x*_, TiO_2_, and Ti_2_CT_*x*_, respectively, at the interface geometries obtained by sp calculations. Both CDD and PA-CDD calculations have been handled with the VASPKIT program.^67^ Later, we integrated the curve^21^ from the PA-CDD plots by means of a homemade python-based program in order to obtain a charge transfer estimation between each building block, Δ*Q*. Note that the separation between the two building blocks was defined as the position at which the PA-CDD changes sign. This point, located in the interface region, provides a physically meaningful criterion to distinguish the charge belonging to the TiO_2_ slabs from that of MXene layers.

## 3 Results and Discussion

### 3.1 TiO_2_/Ti_2_CT_*x*_ (T_*x*_ = None, Cl, F, H, O, and OH) Interfaces

We start by discussing the interaction between TiO_2_ and Ti_2_CT_*x*_ (T_*x*_ = none, Cl, F, H, O, and OH) MXenes. Table 1 reports the interface energy as defined in eq 1 of each heterostructure. When the Ti_2_C­(0001) surface is terminated with T_*x*_ = Cl, F, and O, the interaction between the two systems is mainly held by dispersion forces (*E*_int_ ≈ −0.3 J/m^2^),^21,86,87^ while new chemical bonds are formed when T_*x*_ = none, H, and OH (*E*_int_ < −1.0 J/m^2^). On the other hand, the nature of the TiO_2_ surfaces is less important, as the surface exposure affects the results quantitatively but not qualitatively. These results indicate that the TiO_2_–Ti_2_CT_*x*_ interaction is mainly governed by the MXene functionalization. More in detail, for the bare Ti_2_C, *E*_int_ is −5.26, −6.11, and −7.50 J/m^2^ for A-TiO_2_(001)/Ti_2_C, A-TiO_2_(101)/Ti_2_C, and R-TiO_2_(110)/Ti_2_C, respectively, indicating a strong interaction with titania surfaces. This value can be compared with the corresponding one for T_*x*_ = H and OH which is significantly lower. In these cases, *E*_int_ assumes comparable values (−1.50 J/m^2^ < *E*_int_ < −1.80 J/m^2^). Notice that the influence of TiO_2_ on *E*_int_ is rather small. For instance, when Ti_2_C is functionalized with −H groups, *E*_int_ is −1.66, −1.72, and −1.48 J/m^2^ for A-TiO_2_(001)/Ti_2_CH_2_, A-TiO_2_(101)/Ti_2_CH_2_, and R-TiO_2_(110)/Ti_2_CH_2_, respectively. Similar variations are observed on *E*_int_ when the Ti_2_C­(0001) surface is functionalized with −OH groups, providing *E*_int_ values of −1.70, −1.63, and −1.80 J/m^2^ for A-TiO_2_(001)/Ti_2_C­(OH)_2_, A-TiO_2_(101)/Ti_2_C­(OH)_2_, and R-TiO_2_(110)/Ti_2_C­(OH)_2_, respectively. Note that variations of *E*_int_ values due to the different titania polymorphs and exposed surfaces are more noticeable on TiO_2_/Ti_2_C heterostructures involving the bare Ti_2_C surface, where larger *E*_int_ values provide larger variations, i.e., −4.69, −5.54, and −6.75 J/m^2^ for A-TiO_2_(001)/Ti_2_C, A-TiO_2_(101)/Ti_2_C, and R-TiO_2_(110)/Ti_2_C, respectively. This can be attributed to the strong interaction between the two units.

**Table 1:** Calculated Interface Energies Including Dispersion Corrections, *E*_int_, given in J/m^2^, for the TiO_2_/Ti_2_CT_*x*_ (T_*x*_ = none, Cl, F, H, O, and OH) Heterostructure, the *a* and *b* Lattice Parameters (in Å), and the Angle, γ (deg), between Them for Each Supercell

TiO_2_	Ti_2_CT_*x*_	*E*_int_	*a*	*b*	γ
A-TiO_2_(001)	Ti_2_CCl_2_	–0.35	24.248	11.368	52.87
	Ti_2_CF_2_	–0.41	29.044	22.104	18.95
	Ti_2_CH_2_	–1.66	29.109	22.074	18.99
	Ti_2_CO_2_	–0.48	28.881	21.998	18.98
	Ti_2_C(OH)_2_	–1.70	29.109	22.108	19.03
	Ti_2_C	–5.26	29.391	22.133	18.99
A-TiO_2_(101)	Ti_2_CCl_2_	–0.20	36.755	11.195	34.51
	Ti_2_CF_2_	–0.40	15.080	10.445	90.00
	Ti_2_CH_2_	–1.72	15.068	10.441	90.02
	Ti_2_CO_2_	–0.45	15.056	10.415	90.00
	Ti_2_C(OH)_2_	–1.63	15.134	10.413	90.00
	Ti_2_C	–6.11	15.130	10.400	89.95
R-TiO_2_(110)	Ti_2_CCl_2_	–0.33	11.749	19.634	81.07
	Ti_2_CF_2_	–0.39	29.442	16.114	23.82
	Ti_2_CH_2_	–1.48	26.244	13.303	29.35
	Ti_2_CO_2_	–0.35	26.173	13.262	29.43
	Ti_2_C(OH)_2_	–1.80	26.221	13.328	29.37
	Ti_2_C	–7.50	26.327	13.403	29.26

Figures 2 and S2 show the optimized heterostructures TiO_2_/Ti_2_CT_*x*_, where T_*x*_ = none, H, and OH, focusing on the interface region. In Figure 2, one can see that for TiO_2_/Ti_2_CH_2_ and TiO_2_/Ti_2_C heterostructures, the O atoms of the TiO_2_ surface are in direct contact with the Ti atoms of the MXene surface with an average Ti–O distance of 2.1 Å, which is close to the typical value in Ti–O-based materials, around 1.9–2.0 Å. In the case of TiO_2_/Ti_2_C­(OH)_2_ heterostructures, there is a rather ordered arrangement of hydrogen-bonding-like interactions between the MXene −OH groups and surface O atoms of TiO_2_ surfaces, with an average distance of 1.4 Å, between those of hydrogen bonds (∼1.7 Å) and fully covalent interactions (∼1.0 Å).

**Figure 2:**
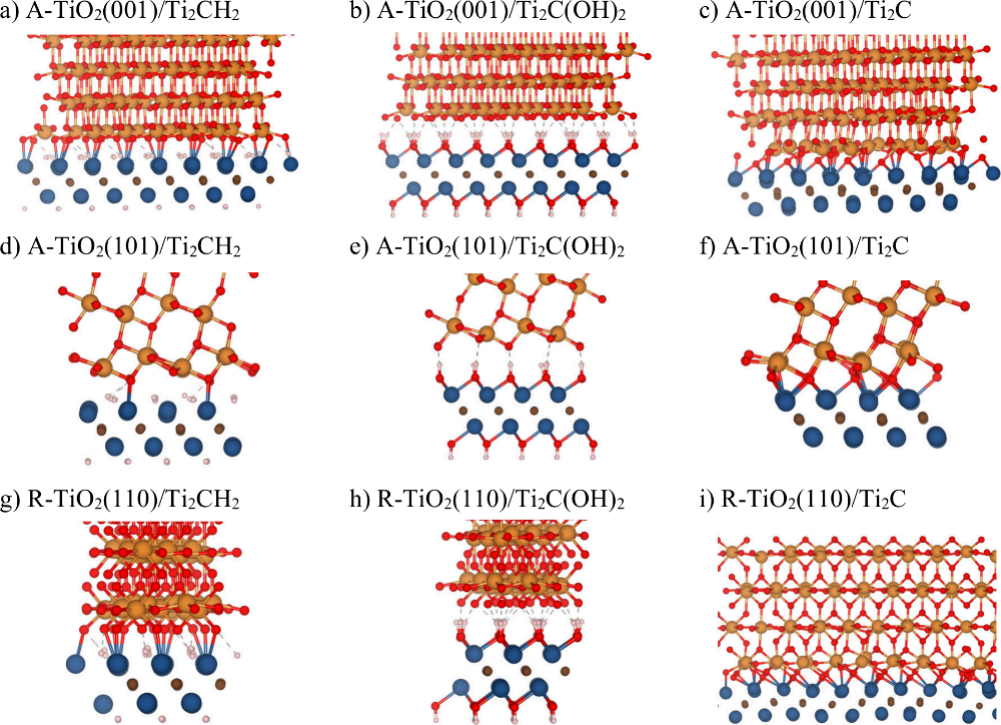
Side views of optimized geometries of (a) A-TiO_2_(001)/Ti_2_CH_2_, (b) A-TiO_2_(001)/Ti_2_C­(OH)_2_, (c) A-TiO_2_(001)/Ti_2_C, (d) A-TiO_2_(101)/Ti_2_CH_2_, (e) A-TiO_2_(101)/Ti_2_C­(OH)_2_, (f) A-TiO_2_(101)/Ti_2_C, (g) R-TiO_2_(110)/Ti_2_CH_2_, (h) R-TiO_2_(110)/Ti_2_C­(OH)_2_, and (i) R-TiO_2_(110)/Ti_2_C. Color-coding as in Figure 1.

### 3.2 Interfacial Polarization

After examining the structure and energetics of interfaces, we now focus on interface polarization. Figure 3 reports the analysis of CDD and PA-CDD for heterostructures with bond formation. In all cases, we observe an electron accumulation on the TiO_2_ phase and an electron depletion on the MXene regardless of the titania polymorph, its exposed surfaces, and the MXene functionalization. More specifically, the electron localization mainly occurs at the interface, with some small propagations through TiO_2_ phases. The integration of the PA-CDD allows us to estimate the charge transfer at the interface, Δ*Q*. The calculated values are reported in Table 2. Focusing on Δ*Q*, the MXene functionalization clearly influences the amount of charge transfer. For instance, Δ*Q* is −0.49, −0.76, and −0.79 *e*/nm^2^ for A-TiO_2_(001)/Ti_2_CH_2_, A-TiO_2_(001)/Ti_2_C­(OH)_2_, and A-TiO_2_(001)/Ti_2_C, respectively.

**Table 2:** Calculated Charge Transfer towards TiO_2_ upon Interaction, Δ*Q*, in *e*/nm^2^ of TiO_2_/Ti_2_CT_*x*_ (T_*x*_ = None, H, and OH) Heterostructures with the HSE06 Functional^a^

TiO_2_	Ti_2_CT_*x*_	Δ*Q*
A-TiO_2_(001)	Ti_2_CH_2_	–0.49
	Ti_2_C(OH)_2_	–0.76
	Ti_2_C	–0.79
A-TiO_2_(101)	Ti_2_CH_2_	–0.50
	Ti_2_C(OH)_2_	–0.94
	Ti_2_C	–1.22
R-TiO_2_(110)	Ti_2_CH_2_	–0.54
	Ti_2_C(OH)_2_	–1.05
	Ti_2_C	–1.43

a The negative sign implies an electron accumulation in the TiO_2_ phase.

**Figure 3:**
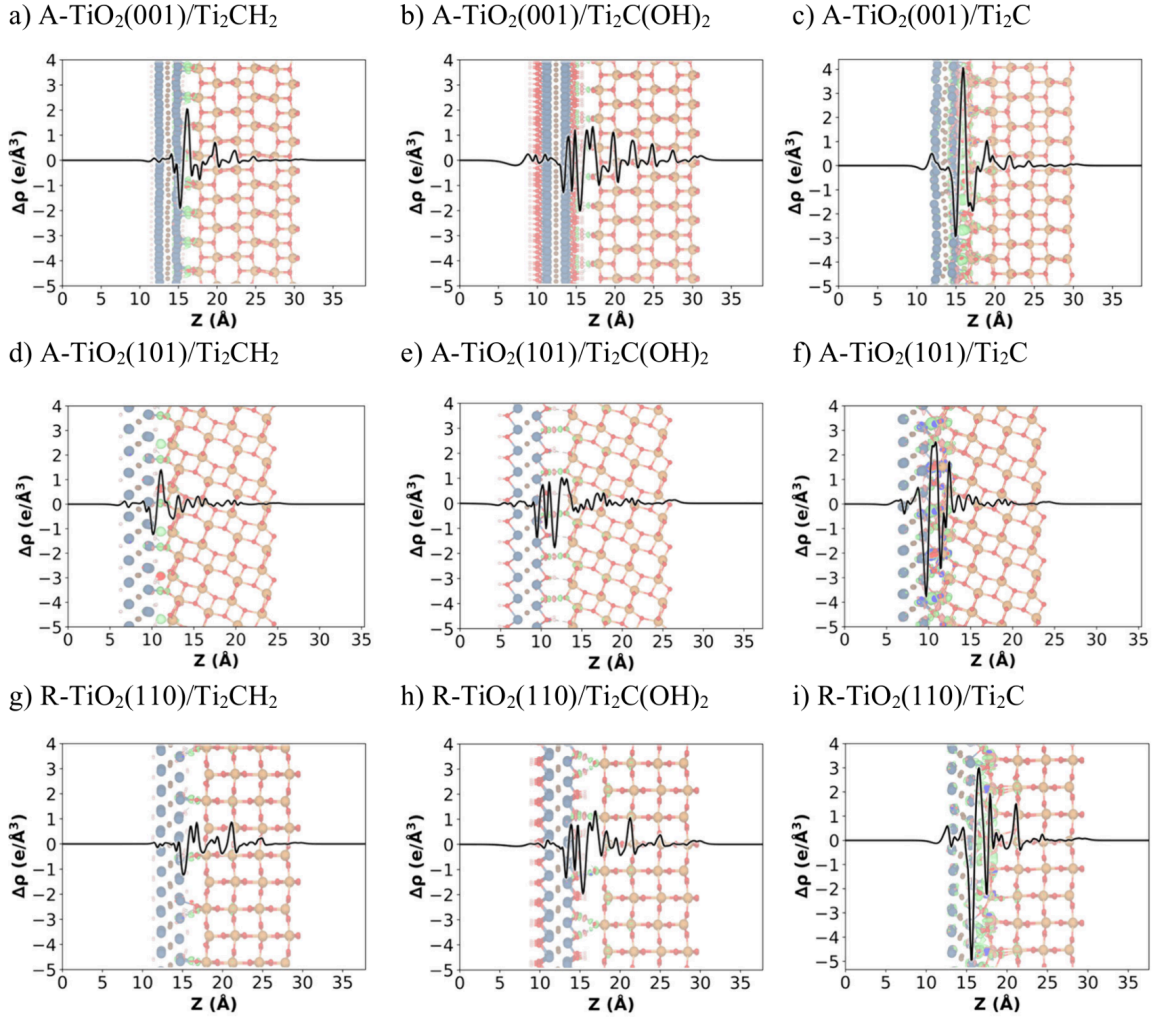
Calculated HSE06 PA-CDD (Δρ in *e*/Å^3^) along the normal direction to the MXene (0001) surface, *z*, for (a) A-TiO_2_(001)/Ti_2_CH_2_, (b) A-TiO_2_(001)/Ti_2_C­(OH)_2_, (c) A-TiO_2_(001)/Ti_2_C, (d) A-TiO_2_(101)/Ti_2_CH_2_, (e) A-TiO_2_(101)/Ti_2_C­(OH)_2_, (f) A-TiO_2_(101)/Ti_2_C, (g) R-TiO_2_(110)/Ti_2_CH_2_, (h) R-TiO_2_(110)/Ti_2_C­(OH)_2_, and (i) R-TiO_2_(110)/Ti_2_C. HSE06 CDD isosurface values of 0.01 *e*/Å^3^ are depicted in the PA-CDD plot, where red and green isosurfaces correspond to depletion and accumulation of electron density, respectively. Color-coding as in Figure 1.

Next, the influence of the TiO_2_ polymorph and its exposed surface is less important in hydrogen-terminated MXene cases, leading to nearly identical charge transfer values of −0.49, −0.50, and −0.54 *e*/nm^2^ for A-TiO_2_(001)/Ti_2_CH_2_, A-TiO_2_(101)/Ti_2_CH_2_, and R-TiO_2_(110)/Ti_2_CH_2_, respectively. The results are different for the strongly interacting interfaces, such as those involving the bare MXene in which Δ*Q* is −0.79, −1.22, and −1.43 *e*/nm^2^ for A-TiO_2_(001)/Ti_2_C, A-TiO_2_(101)/Ti_2_C, and R-TiO_2_(110)/Ti_2_C, respectively. Note that these results are consistent with both PBE and HSE06 functionals, with changes in the calculated charge transfer as high as 0.1 *e*/nm^2^ (see Table S6).

Interestingly, the charge transfer always occurs from the MXene surface toward the TiO_2_ phase, implying that electrons are donated from the metal to the semiconductor, resulting in an interface dipole,^21,88^ in agreement with the qualitative interpretation of the difference in the above-mentioned work functions. The observed charge transfer from MXene to TiO_2_ could arise from the different positioning of the band edges between the two materials upon formation of chemical bonds at the interface. This built-in electric field near the interface could drive the electrons toward metallic MXenes upon a possible photoexcitation, leading to a more efficient separation of the charge carriers.^21^ The Density of States (DOS) of the TiO_2_/MXene (M = Ti) interface is reported in Figure S1. The Fermi level of all MXenes lies above the Valence Band Maximum (VBM) of all TiO_2_ phases (see Figure S1a–f), explaining the difference in the work functions and the direction of the charge transfer. The formation of the metal–semiconductor junction leads to the appearance of MXene states close to the conduction band of TiO_2_. This explains the observed electron transfer from the MXene to TiO_2_, where some empty states of the oxide become filled upon interface formation. It must be mentioned that the presence of defects possibly could have some implications.

In summary, the Ti_2_C­(0001) surface functionalization is the main factor that drives the formation of TiO_2_/MXene heterostructures, with T_*x*_ = none, H, and OH surfaces being the terminations that allow the creation of these interfaces. In fact, the bond formation is always coupled to a systematic charge transfer from MXene surfaces toward the TiO_2_ phases. These findings regarding the interface polarization may help the understanding of the energy level alignments usually carried out in experimental works.

## 4 Conclusions

Here we modeled TiO_2_/Ti_2_CT_*x*_ interfaces by means of DFT calculations, considering a series of effects driving the formation of interfaces, such as the effect of the MXene termination, and the nature and surface exposure of TiO_2_. Our results reveal that the interaction between these systems is mainly governed by the MXene surface functionalization; one can find interfaces held by weak dispersion interactions (*E*_int_ ≈ −0.3 J/m^2^, for T_*x*_ = Cl, F, and O) or interfaces with the two units in intimate contact due to the formation of new chemical bonds (*E*_int_ < −1.0 J/m^2^ for T_*x*_ = none, H, and OH). In just a few cases it is possible to have interfaces where new chemical bonds are formed. The nature of the TiO_2_ phase and surface exposure have smaller effects. The formation of interfaces is always accompanied by a charge transfer from MXene surfaces toward TiO_2_ phases regardless of the MXene functionalization, TiO_2_ polymorph, and its exposed surface. The polarization is mainly localized at the interface. The results of this study suggest that TiO_2_/MXene interfaces can form only if the latter has specific terminations, *i.e.*, −H or −OH, or if it is bare. This will also drive the strength of the interaction, irrespective of the nature of TiO_2_, either A-TiO_2_ or R-TiO_2_. The formation of the interface will also generate an interface dipole with a specific orientation. These results can also correlate the surface termination with the interface properties, an aspect that could be of interest for experimental design of these systems. Our results provide insights into trends that could explain why hydrothermal oxidation is consistently performed in all experiments. This process typically replaces −F and −Cl groups with −OH in most cases or leads to the addition of TiO_2_ in an aqueous medium.^36–41,43^ The reason behind this is that −F and −Cl would not form an interface with new chemical bonds. Finally, we note that even if our results have been obtained for the Ti_2_C MXene, it is likely that the present findings hold for other Ti-based MXenes and, more generally, may help the design of TiO_2_/MXene composites and the fundamental understanding of the behavior of interfaces in catalytic applications.

## Supplementary Material



## Notes

Structural parameters and surface energies (Table S1); strain effect on TiO_2_ phases (Table S2); strain effect on MXene phases (Table S3); effect of k-points (Table S4); effect of spin polarization (Table S5); PBE Δ*Q* (Table S6); total DOS (Figure S1); and side views of all optimized heterostructures (Figure S2) (PDF)
