# Supplementary material for: Functionalization-Driven Formation of TiO2/Ti2CT x Interfaces
Source: J Phys Chem C Nanomater Interfaces. 2025 Oct 17;129(43):19551–9. doi: 10.1021/acs.jpcc.5c04505 (PMC12581151; doi:10.1021/acs.jpcc.5c04505)

Functionalization-Driven Formation of TiO_2/Ti_2CT_x Interfaces

Néstor García-Romeral,[a] Giovanni Di Liberto,[b]* Ángel Morales-García,[a]* Francesc Viñes,[a] Francesc Illas,[a] Gianfranco Pacchioni[b]

[a]*Departament de Ciència de Materials i Química Física & Institut de Química Teòrica i Computacional (IQTCUB), Universitat de Barcelona, c/ Martí i Franquès 1-11, 08028 Barcelona, Spain.*

[b]*Dipartimento di Scienza dei Materiali, Università di Milano–Bicocca, via R. Cozzi 55, 20125, Milano, Italy.*

*E-mails: giovanni.diliberto@unimib.it ; angel.morales@ub.edu

Table of contents

Table S1. Structural parameters and surface energies.

Table S2. Strain effect on TiO_2 phases.

Table S3. Strain effect on MXene phases.

Table S4. Effect of k-points.

Table S5. Effect of spin polarization.

Table S6. PBE ΔQ.

Figure S1. Total DOS.

Figure S2. Side views of all optimized heterostructures.

Table S1. Calculated lattice parameters (*a* and *b*, both in Å) as predicted by the PBE functional, HSE06 estimate of surface energies (γ, in J/m^2), slab thickness (*d*, in Å), and total number of atoms (*Num*) of each optimized unit.

System	*a*	*b*	γ	*d*	*Num*
**A-TiO_2(001)**	3.645	3.852	1.307	12.134	18
**A-TiO_2(101)**	3.758	10.383	0.942	12.921	48
**R-TiO_2(110)**	6.471	2.961	0.895	13.071	24
**Ti_2CCl_2**	3.183	5.573	—	5.502	10
**Ti_2CF_2**	2.979	5.335	—	4.794	10
**Ti_2CH_2**	2.978	5.235	—	4.323	10
**Ti_2CO_2**	3.036	5.221	—	4.415	10
**$Ti_2C(OH)_2$**	3.016	5.328	—	6.778	14
**Ti_2C**	3.033	5.226	—	2.289	6

Table S2. Calculated lattice mismatch in percentage (*a* and *b*), and band gap (E_g) in eV of the isolated TiO_2 phases and with the strain at the interface geometry of each heterostructure as predicted by the PBE functional. *Num* stands for the total number of atoms of each supercell.

System	*a*	*b*	E_g	*Num*
**A-TiO_2(001)**	—	—	1.910	—
**A-TiO_2(001)/Ti_2CCl_2**	2.4%	-0.2%	2.067	413
**A-TiO_2(001)/Ti_2CF_2**	0.7%	0.2%	1.960	400
**A-TiO_2(001)/Ti_2CH_2**	0.0%	0.6%	1.914	400
**A-TiO_2(001)/Ti_2CO_2**	1.3%	0.6%	2.004	400
**A-TiO_2(001)/$Ti_2C(OH)_2$**	0.0%	0.3%	1.914	452
**A-TiO_2(001)/Ti_2C**	-1.4%	1.5%	1.813	348
**A-TiO_2(101)**	—	—	2.365	—
**A-TiO_2(101)/Ti_2CCl_2**	0.7%	-0.3%	2.394	418
**A-TiO_2(101)/Ti_2CF_2**	-0.3%	-0.6%	2.360	292
**A-TiO_2(101)/Ti_2CH_2**	-0.2%	-0.6%	2.365	292
**A-TiO_2(101)/Ti_2CO_2**	-0.1%	-0.3%	2.377	292
**A-TiO_2(101)/$Ti_2C(OH)_2$**	-0.7%	-0.3%	2.360	332
**A-TiO_2(101)/Ti_2C**	-0.7%	-0.2%	2.363	252
**R-TiO_2(110)**	—	—	2.046	—
**R-TiO_2(110)/Ti_2CCl_2**	0.1%	0.8%	2.067	413
**R-TiO_2(110)/Ti_2CF_2**	-0.6%	0.6%	2.032	360
**R-TiO_2(110)/Ti_2CH_2**	-0.2%	1.0%	2.057	321
**R-TiO_2(110)/Ti_2CO_2**	0.0%	1.1%	2.069	321
**R-TiO_2(110)/$Ti_2C(OH)_2$**	-0.5%	1.1%	2.044	363
**R-TiO_2(110)/Ti_2C**	-1.1%	1.1%	2.018	279

Table S3. Calculated lattice parameter mismatch in percentage (*a* and *b*), Fermi energy level (E_F), work function (ϕ) in eV, and the relative stability of MXene phase with the strain at the interface with respect the optimized slab model, ΔE, in eV per Ti_2CT_x unit, *n*, for each Ti_2CT_x slab model with the strain at the interface geometry of each heterostructure as predicted by the PBE functional.

System	*a*	*b*	ϕ	$\Delta E/n$
**Ti_2CCl_2**	—	—	4.486	—
**A-TiO_2(001)/Ti_2CCl_2**	-0.6%	1.6%	4.400	0.019
**A-TiO_2(101)/Ti_2CCl_2**	-2.3%	1.3%	4.427	0.069
**R-TiO_2(110)/Ti_2CCl_2**	-2.5%	-0.1%	4.504	0.072
**Ti_2CF_2**	—	—	5.014	—
**A-TiO_2(001)/Ti_2CF_2**	-2.7%	2.2%	4.947	-0.098
**A-TiO_2(101)/Ti_2CF_2**	-2.1%	2.3%	4.938	0.087
**R-TiO_2(110)/Ti_2CF_2**	-3.1%	2.7%	4.964	0.131
**Ti_2CH_2**	—	—	4.237	—
**A-TiO_2(001)/Ti_2CH_2**	-0.9%	-1.7%	4.160	-0.053
**A-TiO_2(101)Ti_2CH_2**	-0.5%	-1.5%	4.202	0.034
**R-TiO_2(110) /Ti_2CH_2**	-2.9%	-1.0%	4.127	0.132
**Ti_2CO_2**	—	—	5.722	—
**A-TiO_2(001)/Ti_2CO_2**	1.4%	-1.6%	5.605	-0.055
**A-TiO_2(101)/Ti_2CO_2**	1.0%	-0.2%	5.641	0.020
**R-TiO_2(110)/Ti_2CO_2**	-0.2%	-2.1%	5.759	0.077
**$Ti_2C(OH)_2$**	—	—	0.742	—
**A-TiO_2(001)/$Ti_2C(OH)_2$**	-1.9%	1.9%	1.021	-0.071
**A-TiO_2(101)/$Ti_2C(OH)_2$**	-0.5%	2.4%	1.167	0.075
**R-TiO_2(110)/$Ti_2C(OH)_2$**	-2.1%	0.9%	0.664	0.060
**Ti_2C**	—	—	4.471	—
**A-TiO_2(001)/Ti_2C**	2.8%	-1.3%	4.552	-0.098
**A-TiO_2(101)/Ti_2C**	1.6%	1.0%	4.490	0.087
**R-TiO_2(110)/Ti_2C**	-3.0%	-4.5%	4.384	0.315

Table S4. PBE calculated total energies, in eV, of the *c*(1×1) Ti_2C cell employing different **k**-points.

k-points	*E*
**1×1×1**	-50.92
**2×2×1**	-52.23
**3×3×1**	-51.64
**4×4×1**	-51.83
**5×5×1**	-51.72

Table S5. Calculated interface energies including dispersion corrections, E_{int}, given in J/m^2, and obtained from calculations with the HSE06 functional of A-TiO_2(101)/Ti_2C heterostructure with open and close-shell approaches of the electronic density.

**Approach**	$\boldsymbol{E_{int}}$
**Closed-shell**	-6.11
**Open-shell**	-6.04

Table S6. Calculated charge transfer towards TiO_2 upon interaction, ΔQ, in e/nm^2 of each TiO_2/Ti_2CT_x (T_x = none, H, and OH) heterostructure with PBE functional. The negative sign implies an electron accumulation in TiO_2 phase.

**TiO_2**	**Ti_2CT_x**	**ΔQ**
**A-TiO_2(001)**	**Ti_2CH_2**	-0.48
	**$Ti_2C(OH)_2$**	-0.84
	**Ti_2C**	-0.80
**A-TiO_2(101)**	**Ti_2CH_2**	-0.49
	**$Ti_2C(OH)_2$**	-0.93
	**Ti_2C**	-1.18
**R-TiO_2(110)**	**Ti_2CH_2**	-0.50
	**$Ti_2C(OH)_2$**	-1.03
	**Ti_2C**	-1.34

Figure S1. HSE06 Total DOS in a.u. for (a) A-TiO_2(001), (b) A-TiO_2(101), (c) R-TiO_2(110), (d) Ti_2C, (e) Ti_2CH_2, and (f) $Ti_2C(OH)_2$ and HSE06 total DOS and Projected DOS an a.u. decomposed per building block contributions for (g) A-TiO_2(001)/Ti_2C, (h) A-TiO_2(101)/Ti_2C, (i) R-TiO_2(110)/Ti_2C, (j) A-TiO_2(001)/Ti_2CH_2, (k) A-TiO_2(101)/Ti_2CH_2, (l) R-TiO_2(110)/Ti_2CH_2, (m) A-TiO_2(001)/$Ti_2C(OH)_2$, (n) A-TiO_2(101)/$Ti_2C(OH)_2$, and (o) R-TiO_2(110)/$Ti_2C(OH)_2$. The energies, E in eV, are referenced to the vacuum level, E_{vac}, while the Fermi level is marked with a dashed line.

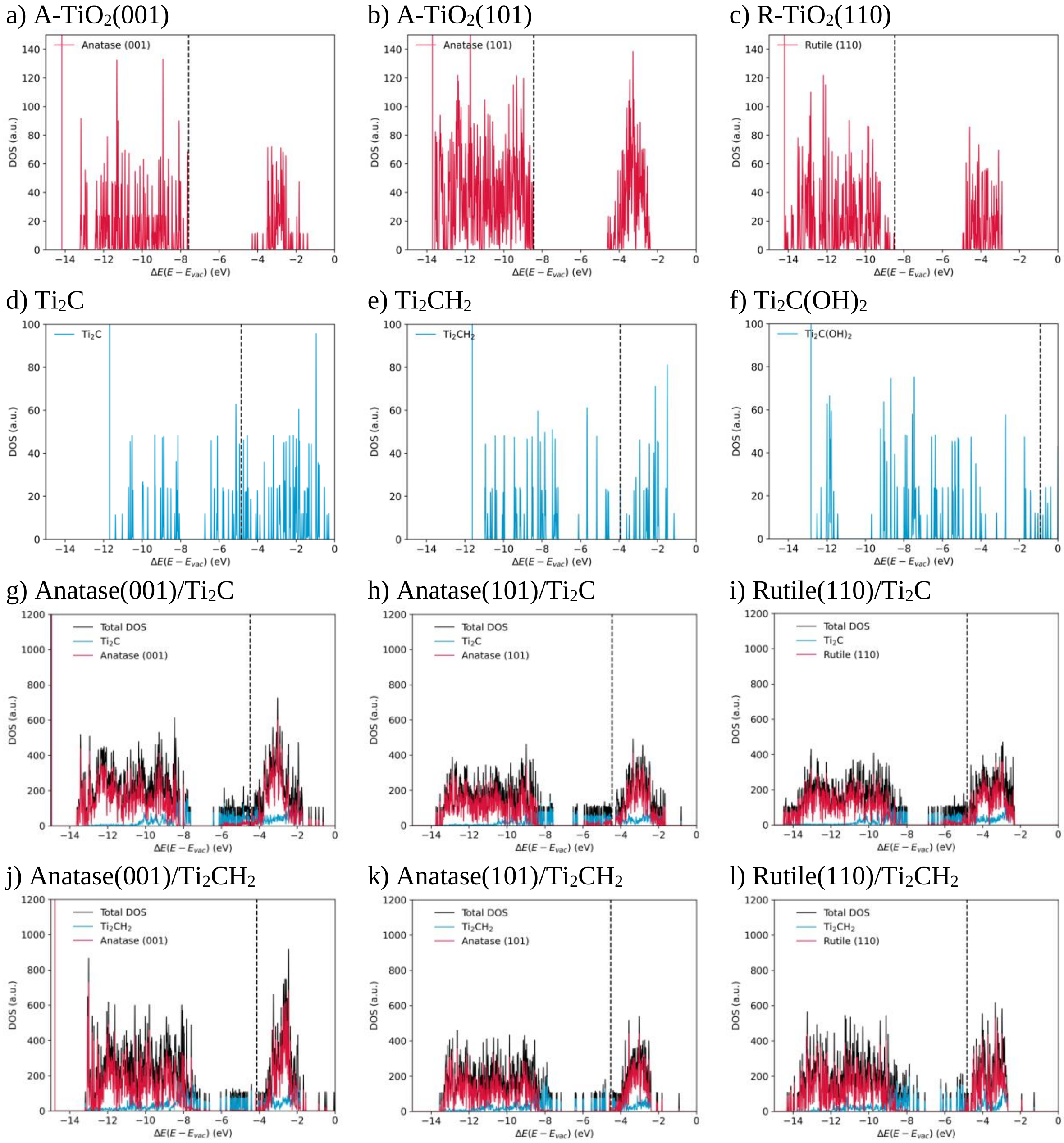

m) Anatase(001)/$Ti_2C(OH)_2$

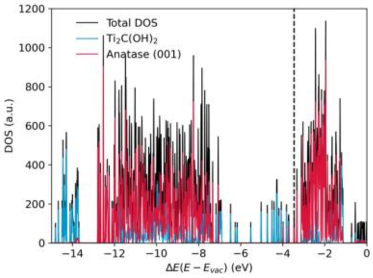

n) Anatase(101)/$Ti_2C(OH)_2$

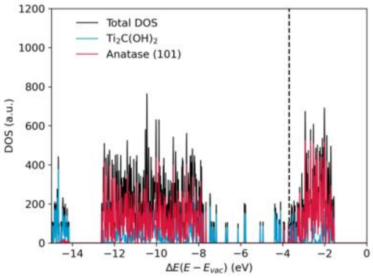

o) Rutile(110)/$Ti_2C(OH)_2$

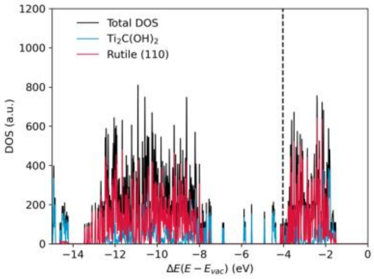

Figure S2. Side view of optimized geometries of (a) A-TiO_2(001)/Ti_2CCl_2, (b) A-TiO_2(001)/Ti_2CF_2, (c) A-TiO_2(001)/Ti_2CO_2, (d) A-TiO_2(101)/Ti_2CCl_2, (e) A-TiO_2(101)/Ti_2CF_2, (f) A-TiO_2(101)/Ti_2CO_2, (g) R-TiO_2(110)/Ti_2CCl_2, (h) R-TiO_2(110)/Ti_2CF_2, (i) R-TiO_2(110)/Ti_2CO_2, (j) A-TiO_2(001)/Ti_2CH_2, (k) A-TiO_2(001)/$Ti_2C(OH)_2$, (l) A-TiO_2(001)/Ti_2C, (m) A-TiO_2(101)/Ti_2CH_2, (n) A-TiO_2(101)/$Ti_2C(OH)_2$, (o) A-TiO_2(101)/Ti_2C, (p) R-TiO_2(110)/Ti_2CH_2, (q) R-TiO_2(110)/$Ti_2C(OH)_2$, and (r) R-TiO_2(110)/Ti_2C. Colour-coding as in Figure 1 of the main text, being the green and purple spheres Cl and F atoms, respectively.

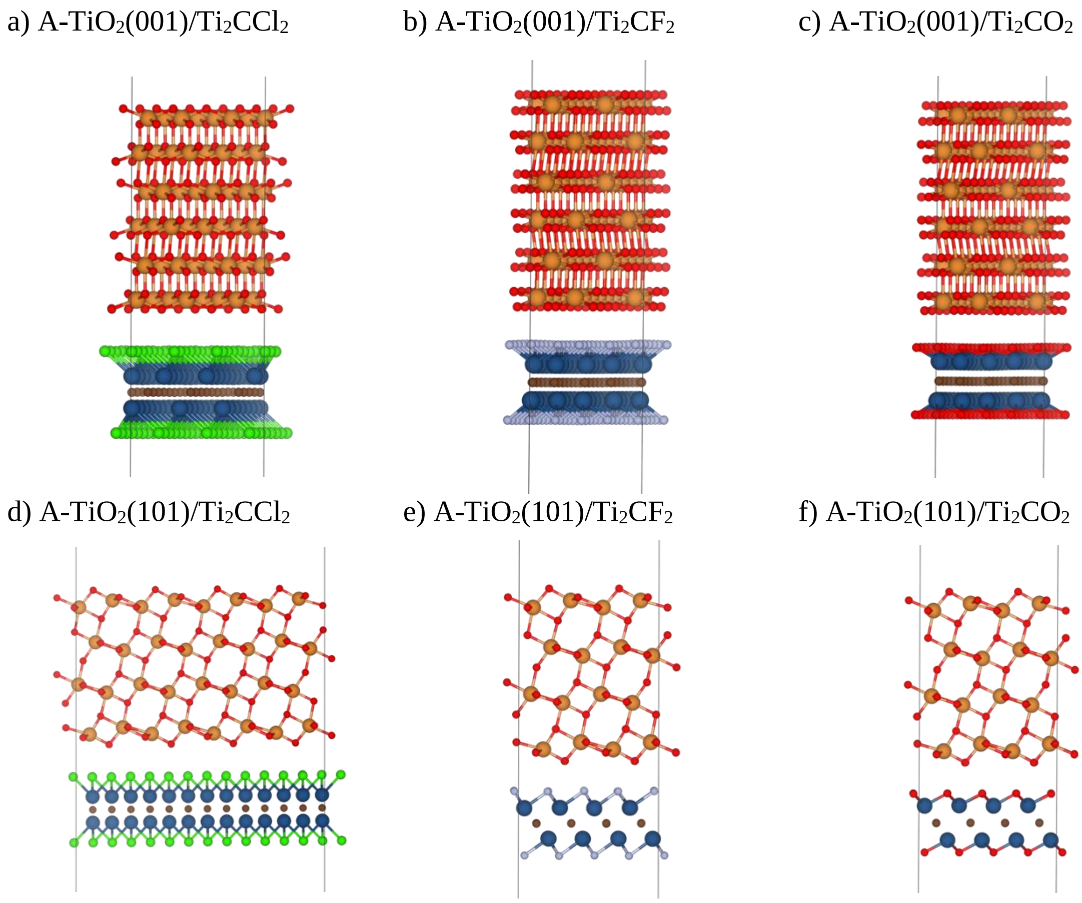

g) R-TiO_2(110)/Ti_2CCl_2

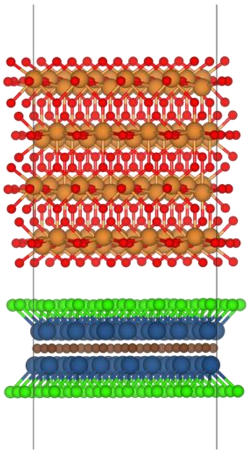

h) R-TiO_2(110)/Ti_2CF_2

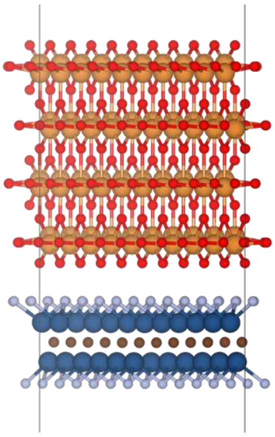

i) R-TiO_2(110)/Ti_2CO_2

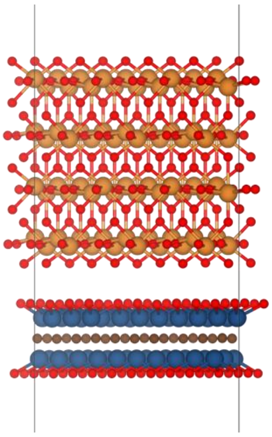

j) A-TiO_2(001)/Ti_2CH_2

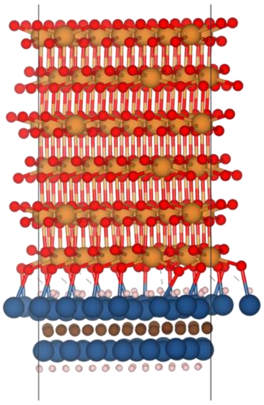

k) A-TiO_2(001)/$Ti_2C(OH)_2$

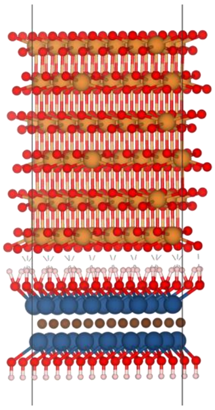

l) A-TiO_2(001)/Ti_2C

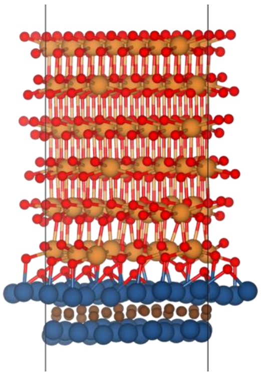

m) A-TiO_2(101)/Ti_2CH_2

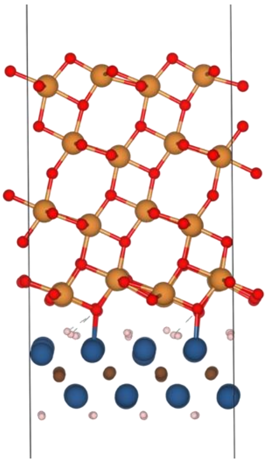

n) A-TiO_2(101)/$Ti_2C(OH)_2$

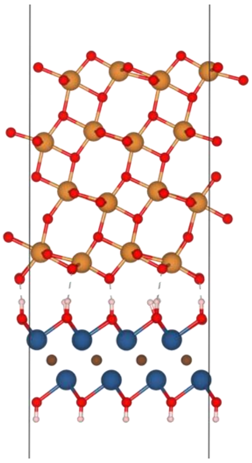

o) A-TiO_2(101)/Ti_2C

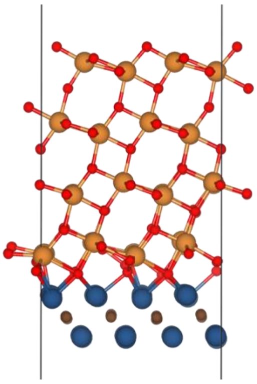

p) R-TiO_2(110)/Ti_2CH_2

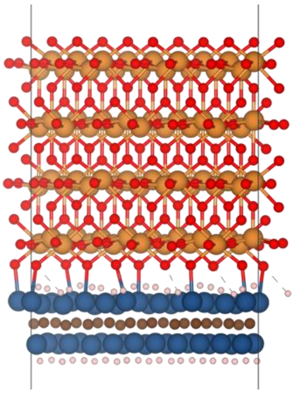

q) R-TiO_2(110)/$Ti_2C(OH)_2$

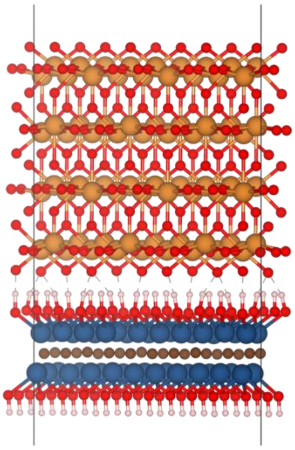

r) R-TiO_2(110)/Ti_2C

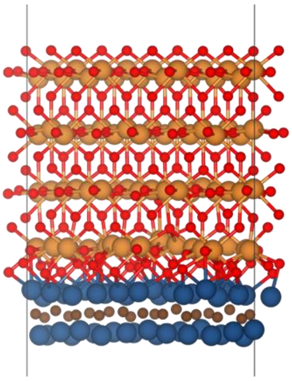

Supplement: Supplementary file 1 [file jp5c04505_si_001.pdf]
